# Depressive Symptoms, Sleep Quality, and Anhedonia in Patients with Primary Biliary Cholangitis: A Cross-Sectional Study

**DOI:** 10.3390/jcm15207967

**Published:** 2026-10-15

**Authors:** Mehmet Ali Saruhan, Fırat Oyman, Berkay Dertsiz, Kemal Saruhan, Gupse Adali

**Affiliations:** 1 Ümraniye Eğitim ve Araştırma Hastanesi, Ümraniye 34760, Turkeygupseadali@gmail.com (G.A.); 2 Department of Child and Adolescent Psychiatry, Faculty of Medicine, Hacettepe University Hospital, Ankara 06100, Turkey

**Keywords:** primary biliary cholangitis, depressive symptoms, sleep quality, anhedonia, liver stiffness measurement

## Abstract

**Background and aim:** This study assessed depressive symptoms, sleep quality, and anhedonia in patients with primary biliary cholangitis (PBC), compared them with healthy controls (HCs), and explored their associations with UDCA treatment response, established prognostic scores, and liver stiffness measurement (LSM) in patients with PBC. **Methods:** In this single-center cross-sectional study, 103 patients with PBC and 50 HCs were evaluated using three validated patient-reported outcome (PRO) instruments: the Beck Depression Inventory (BDI), Pittsburgh Sleep Quality Index (PSQI), and Snaith–Hamilton Pleasure Scale (SHAPS). PRO measures were compared between patients with PBC and HCs, and associations between PROs and UDCA treatment response, prognostic scores, and LSM were explored in the PBC group. **Results:** Moderate-to-severe depressive symptoms, poor sleep quality, and anhedonia were observed in 45%, 62%, and 36% of patients with PBC, respectively, compared with 18%, 38%, and 18% of HCs. The age- and sex-adjusted odds ratios (ORs) for PBCvs. HC were 3.889 (95% CI 1.695–8.926), 3.442 (95% CI 1.636–7.238), and 2.678 (95% CI 1.158–6.193), respectively. Younger age was independently associated with poor sleep quality (OR per year 0.946, 95% CI 0.898–0.997; *p* = 0.039). No statistically significant associations were identified between PROs and UDCA response, prognostic scores, and LSM in patients with PBC. **Conclusions:** Patients with PBC reported a substantial and interrelated burden of depressive symptoms, poor sleep quality, and anhedonia compared with HCs.

## 1. Introduction

Primary biliary cholangitis (PBC) is a chronic, progressive cholestatic liver disease characterized by immune-mediated destruction of intrahepatic bile ducts, ultimately leading to fibrosis, cirrhosis, and liver failure if untreated. Although early diagnosis and treatment, particularly with ursodeoxycholic acid (UDCA), have significantly improved transplant-free survival, a substantial proportion of patients continue to experience a persistent symptom burden despite an adequate biochemical response [1].

Fatigue, depressive symptoms, and sleep disturbances are among the most frequently reported and clinically impactful symptoms in patients with PBC, all of which contribute substantially to impaired health-related quality of life (HRQOL) [2]. While these symptoms have been described predominantly in European and North American cohorts, data from other populations are limited. Cultural differences, healthcare systems, symptom perception, and disease characteristics, including treatment response and disease stage at presentation, may influence patient-reported outcomes (PROs), highlighting the need for population-specific data on PROs.

The assessment of HRQOL in PBC has substantially advanced with the development of disease-specific instruments, such as the PBC-40, which enables a multidimensional evaluation of symptom burden [3]. However, while the PBC-40 captures key symptom domains, it does not directly quantify specific neuropsychiatric constructs, such as depression severity or anhedonia, nor does it provide a detailed assessment of sleep quality in patients with PBC. Consequently, important dimensions of PROs may remain insufficiently characterized in the literature. Validated neuropsychiatric instruments, such as the Beck Depression Inventory (BDI), Pittsburgh Sleep Quality Index (PSQI), and Snaith–Hamilton Pleasure Scale (SHAPS), may provide complementary information on the psychological burden of PBC. In particular, anhedonia, a core feature of depression that may be mistaken for fatigue, has received little attention in patients with PBC.

To date, comprehensive studies simultaneously evaluating depressive symptoms, sleep quality, and anhedonia in patients with PBC are scarce, and data from Türkiye are lacking. Furthermore, it remains uncertain whether these psychological symptoms are associated with liver disease severity, the biochemical response to UDCA, or established prognostic scores.

Therefore, this study aimed to evaluate depressive symptoms, sleep disturbances, and anhedonia using validated PROs in Turkish patients with PBC and to compare these findings with those of healthy control subjects. In addition, we examined the relationships between these neuropsychiatric symptoms and clinical characteristics, biochemical disease severity, UDCA treatment response, and established prognostic scores.

## 2. Methods

### 2.1. Study Participants

This single-center cross-sectional study was conducted at the adult hepatology outpatient clinic of Ümraniye Training and Research Hospital, Istanbul, Türkiye, between February and June 2025. Patients regularly followed up for PBC were consecutively screened. PBC was diagnosed according to the EASL criteria using clinical, biochemical, serological, and, when available, histological findings [4]. Inclusion criteria were confirmed PBC, age > 18 years, UDCA treatment for at least 1 year, and willingness and ability to complete the questionnaires. Exclusions included other chronic liver diseases, major depressive disorder, inability to complete the questionnaires, current psychotropic and centrally acting medication use, and ascites. Concomitant MASLD was included. Healthy controls (HCs) were >18 years hospital staff, patient relatives, and other volunteers recruited at the institution during the same period. The absence of known chronic medical conditions, liver disease, and major depressive disorder was assessed through history taking and review of available medical records. HCs were selected to approximate the patient age and sex distribution; however, the final groups differed significantly in age.

During regular follow-up visits, data were prospectively collected using an electronic case report form. The dataset included demographic and clinical variables (age, sex, body mass index [BMI], disease duration, and current therapy), laboratory parameters obtained within 4 weeks of assessment, the presence of metabolic dysfunction-associated steatotic liver disease (MASLD), and clinical features of chronic liver disease, including the presence and severity of ascites and encephalopathy, and a history of variceal bleeding. In addition, the biochemical response to ursodeoxycholic acid therapy was assessed according to the Paris II criteria and prognosis using the GLOBE score [5]. MASLD was defined as the presence of hepatic steatosis, in addition to at least one of the five cardiometabolic risk factors according to established criteria [6]. All patients underwent vibration-controlled transient elastography (VCTE) using a FibroScan^®^ 530 Compact (Echosens, Paris, France) on the same day as clinical evaluation. Measurements were performed after a minimum fasting period of 4 h by two experienced operators, and the controlled attenuation parameter (CAP) and liver stiffness measurement (LSM) values were recorded. Hepatic steatosis was defined as a controlled attenuation parameter (CAP) ≥248 dB/m, and LSM ≥8 kPa was used as an elevated liver stiffness category [7]. The PBC-specific thresholds of 8 and 15 kPa distinguish prognostic risk groups [8]. Cirrhosis was defined clinically based on a combination of laboratory and radiological findings consistent with advanced chronic liver disease and/or portal hypertension; histological confirmation was not required. Child–Pugh and MELD scores were recorded in patients with cirrhosis.

All patients and HCs were informed of the study’s aims, and written informed consent was obtained from all participants prior to enrolment and questionnaire completion. This study was approved by the Ethics Committee of Ümraniye Training and Research Hospital (approval no. E-54132726-000-265875018). The study was conducted in accordance with the Declaration of Helsinki and its amendments.

### 2.2. Questionnaires

Depressive symptoms were assessed using the Beck Depression Inventory (BDI), a 21-item self-report questionnaire originally developed by Beck et al. and validated in the Turkish population [9,10]. Each item is scored on a 4-point Likert scale ranging from 0 to 3, yielding a total score between 0 and 63, with higher scores indicating greater severity of depressive symptoms. The BDI scores were categorized as follows: 0–9 (minimal depressive symptoms), 10–18 (mild), 19–29 (moderate), and ≥30 (severe depressive symptoms) [9,11]. Moderate-to-severe depressive symptoms were defined as BDI scores ≥19. No structured psychiatric interview was performed.

Sleep quality was assessed using the Pittsburgh Sleep Quality Index (PSQI), a validated self-report questionnaire that evaluates sleep quality over the preceding month across seven domains: subjective sleep quality, sleep latency, sleep duration, habitual sleep efficiency, sleep disturbances, use of sleep medication, and daytime dysfunction. The global PSQI scores ranged from 0 to 21, with higher scores indicating a poorer sleep quality. A global score of >5 was considered indicative of clinically significant sleep disturbance. The Turkish version of the PSQI has demonstrated good validity and reliability [12,13].

Anhedonia was assessed using the Snaith–Hamilton Anhedonia Scale (SHAPS), a 14-item self-report instrument developed to evaluate hedonic capacity. Each item was rated in a dichotomous format, with higher scores indicating greater anhedonia. The Turkish version of the SHAPS has previously demonstrated satisfactory validity and reliability. In the present study, a score ≥ 3 was considered indicative of clinically relevant anhedonia [14,15].

### 2.3. Statistical Analysis

Statistical analyses were performed using the SPSS Statistics software (version 31.0.2.0; IBM Corp., Armonk, NY, USA). Continuous variables with a normal distribution are presented as mean ± standard deviation (SD), whereas variables with a non-normal distribution are expressed as medians (minimum–maximum). The normality of the distribution was assessed using the Kolmogorov–Smirnov test.

Comparisons between groups were conducted using the independent samples t-test (Welch’s test for the age comparison between patients and controls) for normally distributed variables and the Mann–Whitney U test for non-normally distributed variables. Categorical variables are presented as numbers and percentages, and comparisons between groups were performed using the chi-square test or Fisher’s exact test when appropriate. A two-tailed *p*-value of <0.05 was considered statistically significant.

Spearman’s correlation analysis evaluated the associations between questionnaire scores and clinical parameters. In binary logistic regression, the event was coded as moderate-to-severe depressive symptoms (BDI ≥ 19), poor sleep quality (PSQI > 5), or anhedonia (SHAPS ≥ 3). Reference categories were female sex, absence of PBC, Paris II response, CAP < 248 dB/m, and LSM < 8 kPa; continuous-variable ORs were reported per unit increase. Predictors with univariable *p* < 0.250 were included in each multivariable model. When only one predictor qualified, a separate multivariable model was not reported. PBC vs. HC comparisons were adjusted for age and sex because these demographic variables could confound the comparisons. Additional exploratory linear models examined continuous BDI, PSQI, and SHAPS scores, adjusted for age and sex, using heteroscedasticity-robust (HC3) standard errors. Within the PBC group, each severity marker was evaluated in a separate age- and sex-adjusted continuous-score model. Effect estimates are reported with 95% confidence intervals.

## 3. Results

### 3.1. Characteristics of Participants

Of the 140 patients screened, 37 were excluded: 15 declined participation, 12 had incomplete questionnaires, four had psychiatric disorders, four had received UDCA for <1 year, and two had ascites. The final cohort comprised 103 patients with PBC and 50 HCs. Patients with PBC were older than HCs (57.2 ± 12.3 vs. 53.5 ± 8.4 years; *p* = 0.032). Sex distribution and BMI did not differ significantly (male sex: 11 [10.7%] vs. 4 [8.0%], *p* = 0.775; BMI: 28.5 ± 4.8 vs. 27.4 ± 5.2 kg/m^2^, *p* = 0.189). Clinical cirrhosis was present in 12 patients (11.7%), and the median MELD score was 8 (IQR 6.75–10.25) and the median Child–Pugh score was 5 (IQR 5–6); none had hepatic encephalopathy; baseline albumin was median 4.05 g/dL (IQR 3.93–4.18). MASLD was present in 26 patients (25.2%). The mean CAP was 224 ± 45 dB/m and the median LSM was 7.6 kPa (range 2.8–73.9). Diabetes and hypertension were present in 23 (22.3%) and 41 (39.8%) patients, respectively, and 12 (11.7%) had ≥3 cardiometabolic risk factors. The mean GLOBE score was −0.43 ± 0.84 (*n* = 98), and 17 patients (17.2%) were classified as high risk. Paris II non-response was recorded in 33 patients (32.0%), and the mean disease duration was 73.6 ± 40.3 months (Table 1).

### 3.2. Comparison of BDI, PSQI and SHAPS Scores Between PBC and Healthy Controls

**Figure 1:**
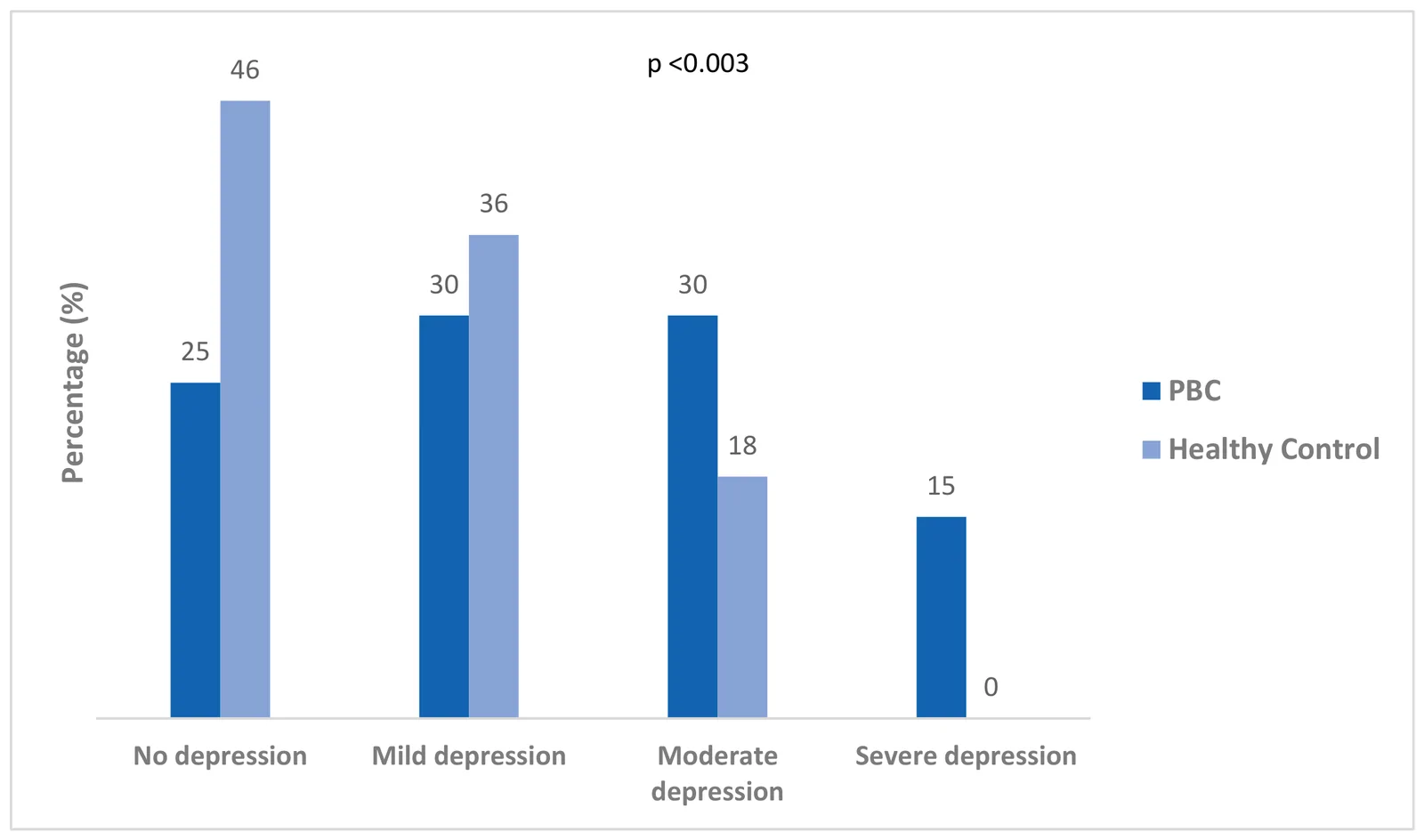
Distribution of depressive symptom severity according to Beck Depression Inventory (BDI) categories in patients with primary biliary cholangitis and healthy controls.

**Figure 2:**
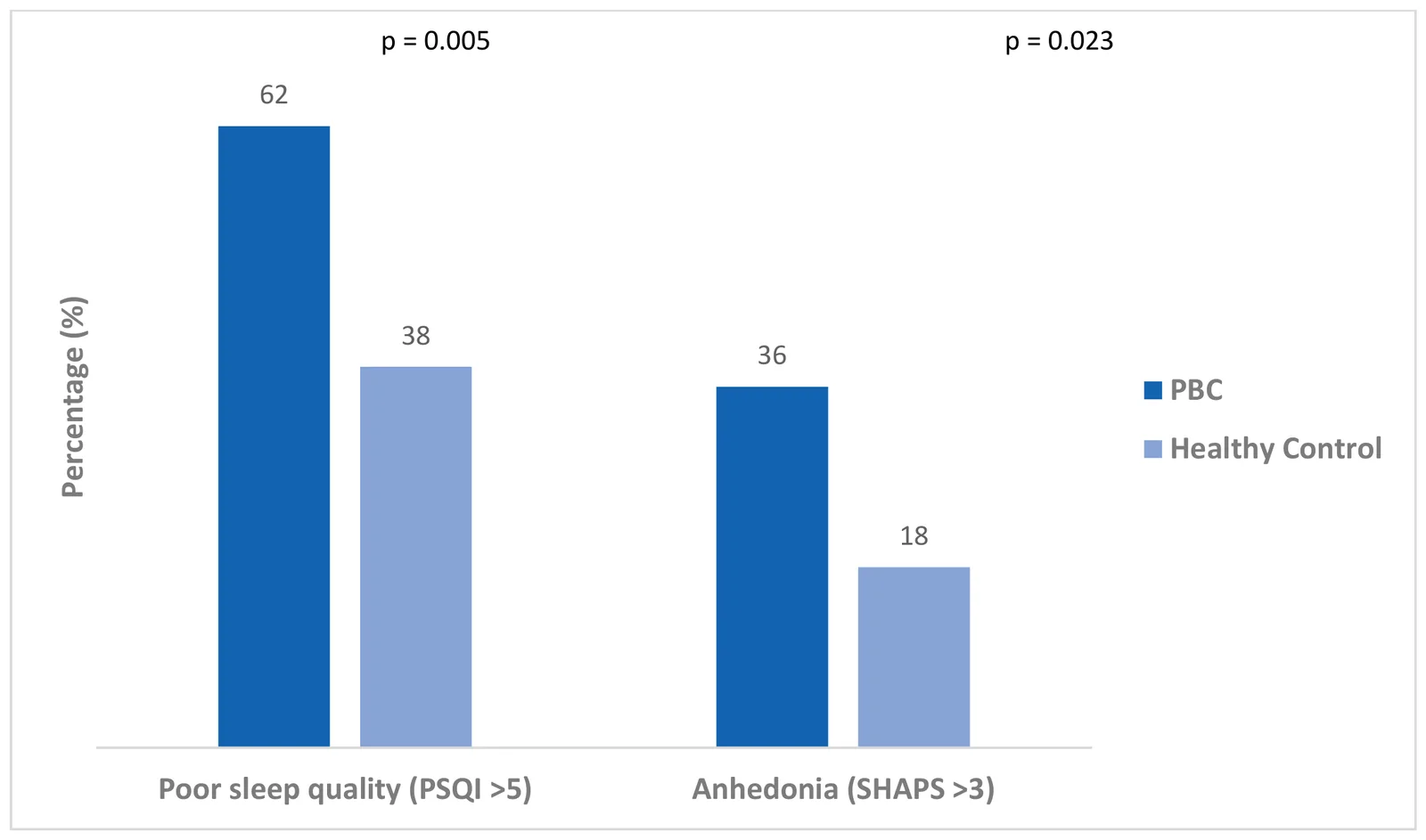
Comparison of poor sleep quality and anhedonia between patients with primary biliary cholangitis and healthy controls.

The median BDI score was 16 (0–45) in patients with PBC and 10 (1–29) in HCs (*p* < 0.001); the median PSQI score was 7 (0–15) in patients with PBC and 4 (1–12) in HCs (*p* = 0.013); and the median SHAPS score was 1 (0–14) in patients with PBC and 0 (0–12) in HCs (*p* = 0.039). When BDI scores were categorized into four categories (no, mild, moderate, and severe depressive symptoms), depressive symptom severity was significantly higher in patients with PBC than in healthy controls (25% vs. 46%, 30% vs. 36%, 30% vs. 18%, and 15% vs. 0%, respectively) (*p* = 0.003) (Figure 1). The use of a cutoff of >5 for poor PSQI sleep quality was significantly more common in patients with PBC (62%) than in HCs (38%), indicating that poor sleep quality and increased sleep disturbance were more prevalent in the PBC group (*p* = 0.005). Using a SHAPS cutoff value of ≥3, clinically relevant anhedonia was observed in 36% of patients with PBC compared to 18.0% of HCs, indicating a significantly higher burden of anhedonia in the PBC group (*p* = 0.023) (Figure 2). After adjusting for age and sex, PBC was associated with moderate-to-severe depressive symptoms (OR 3.889, 95% CI 1.695–8.926; *p* = 0.001), poor sleep quality (OR 3.442, 95% CI 1.636–7.238; *p* = 0.001), and anhedonia (OR 2.678, 95% CI 1.158–6.193; *p* = 0.021) (Table S4). Exploratory continuous-score models estimated adjusted PBC-versus-control differences of 6.370 BDI points (95% CI 3.174–9.565; *p* < 0.001; *n* = 139), 1.823 PSQI points (95% CI 0.778–2.869; *p* < 0.001; *n* = 140), and 1.205 SHAPS points (95% CI 0.253–2.157; *p* = 0.013; *n* = 140) (Table S5).

### 3.3. Comparison of Clinical Characteristics of Patients with PBC According to BDI, PSQI and SHAPS Categories

Patients were categorized into those with moderate-to-severe depressive symptoms (*n* = 46, 45%) and those with minimal or mild depressive symptoms (*n* = 57, 55%). There were no significant differences between the two groups in terms of age, sex, or BMI. Similarly, the prevalence of hepatic steatosis, elevated liver stiffness (LSM ≥ 8 kPa), MASLD, and cardiometabolic parameters were comparable between the groups. The GLOBE score, Paris II non-response, and disease duration were also similar between the groups (Supplementary Table S1). Of the 103 patients with PBC, 64 (62%) had poor sleep quality and 39 (38%) had good sleep quality. Patients with poor sleep quality were younger (54.9 ± 12.9 vs. 60.9 ± 10.3 years) than those with good sleep quality (*p* = 0.017). No significant differences were observed between the groups in terms of sex distribution or BMI (*p* = 0.243). No significant associations were observed between sleep quality and other clinical parameters (Supplementary Table S2). Anhedonia was present in 37 patients (36%) and was not observed in 66 patients (64%). No significant differences were observed between the groups in terms of age, sex, or BMI (*p* = 0.582). Other disease characteristics were also similar between patients with and without anhedonia, including the GLOBE score, Paris II response, and disease duration. The prevalence of metabolic comorbidities, including diabetes and hypertension, was also comparable (Supplementary Table S3).

### 3.4. Correlation of BDI, PSQI and SHAPS Scores with Clinical Parameters in Patients with PBC

**Figure 3:**
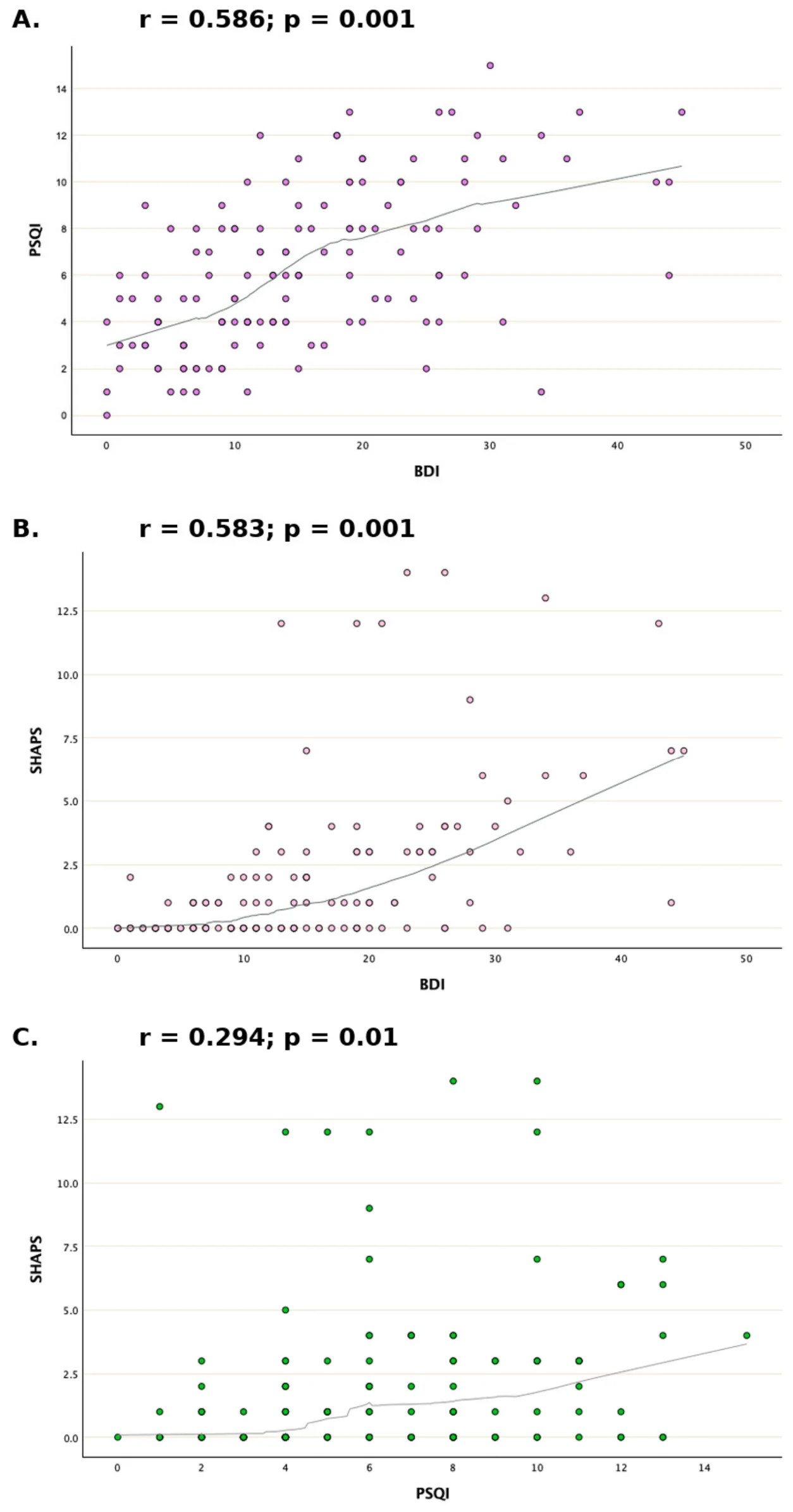
Correlation between BDI, PSQI, and SHAPS scores in patients with PBC. (**A**) Correlation between BDI and PSQI scores (**B**) Correlation between BDI and SHAPS scores (**C**) Correlation between PSQI and SHAPS scores. Each dot represents an individual patient observation, and the lines represent fitted trend lines.

A positive correlation was observed between BDI and PSQI scores (r = 0.586, *p* < 0.001). Similarly, BDI scores were positively correlated with SHAPS scores (r = 0.583, *p* < 0.001), indicating that greater depressive symptom severity was associated with higher anhedonia levels. In addition, PSQI scores were positively correlated with SHAPS scores (r = 0.294, *p* = 0.005), suggesting that impaired sleep quality is associated with increased anhedonia (Figure 3). 

A significant negative correlation was observed between PSQI score and age (r = −0.281, *p* = 0.007), indicating that younger patients tended to have poorer sleep quality. No significant correlations were identified between the BDI, PSQI, or SHAPS scores and other clinical parameters, including the GLOBE score, CAP score, LSM, disease duration, alkaline phosphatase level, total bilirubin level, and BMI.

### 3.5. Logistic Regression Analyses of Factors Associated with Moderate-to-Severe Depressive Symptoms, Poor Sleep Quality, and Anhedonia

In the logistic regression analysis evaluating factors associated with moderate-to-severe depressive symptoms, male sex, liver stiffness measurement (LSM) ≥8 kPa, and disease duration met the predefined thresholds for inclusion in the multivariate model in the univariate analysis. However, none of these variables remained independently associated with moderate-to-severe depressive symptoms in multivariate analysis (Table 2).

Poor sleep quality was coded as an event, and increasing age was associated with lower odds of poor sleep in the univariable model (OR per year 0.958, 95% CI 0.925–0.993; *p* = 0.020). Age, GLOBE score, and disease duration met the *p* < 0.250 selection criterion. In the multivariable model including these three predictors, the age OR was 0.946 (95% CI 0.898–0.997; *p* = 0.039). The GLOBE score and disease duration were not statistically significant (Table 3).

No evaluated predictor was significantly associated with anhedonia in the univariate logistic regression. Male sex was the only predictor meeting the *p* < 0.250 criterion (OR 0.362, 95% CI 0.074–1.773; *p* = 0.210). As a model containing this predictor alone would reproduce the univariate analysis, no separate multivariate model was presented (Table 4). Additional age- and sex-adjusted continuous-score analyses detected no statistically significant associations with LSM, clinical cirrhosis, GLOBE score, Paris II non-response, ALP, or total bilirubin (Tables S6–S8).

## 4. Discussion

We evaluated patient-reported outcomes (PROs) related to depressive symptoms, sleep quality, and anhedonia in patients with PBC. Depressive symptoms and sleep problems have been associated with impaired HRQOL in patients with PBC, while fatigue, closely related to anhedonia, has been linked to reduced quality of life [16]. In the present study, depressive symptoms, poor sleep quality, and anhedonia were significantly more prevalent in patients with PBC than in the HCs. We identified significant correlations among the BDI, PSQI, and SHAPS scores, suggesting that these domains represent the interconnected components of the neuropsychiatric burden in patients with PBC. However, no statistically significant associations were detected between PRO measurements and UDCA treatment response, prognostic scores, or LSM in patients with PBC.

Depressive symptoms appear to be an important component of the overall symptom burden in patients with PBC. In our cohort, moderate-to-severe depressive symptoms, as assessed using the BDI, were observed in 45% of patients with PBC, compared with 18% of healthy controls. This finding is broadly consistent with the literature, suggesting that depressive symptoms are common in PBC and contribute substantially to impaired HRQOL. In studies of patients with PBC with PROs, the prevalence of depression ranged between 28% and 53% [17,18,19,20,21]. Moreover, our findings indicate a substantially greater burden of depressive symptoms than that reported in the general Turkish population. In the nationally representative Türkiye Health Survey of 34,326 adults, the prevalence of probable major depression was reported as 6.3% overall and 8.0% among women based on a validated self-report depression measure, indicating that the psychological burden observed in our PBC cohort was greater than that in the general population [22].

Although moderate-to-severe depressive symptoms were identified in 45% of our PBC cohort, this finding should be interpreted cautiously, as depressive symptom scores in PBC may partly reflect an overlap with other burdensome disease-related symptoms, such as fatigue and pruritus, both of which are well-recognized contributors to impaired PROs and HRQOL. Disease-specific instruments, such as the PBC-40 and CLDQ-PBC, were specifically developed to capture the multidimensional symptom burden of PBC, including fatigue, itch, emotional well-being, sleep disturbance, and social functioning, and may therefore provide a more comprehensive assessment of overall HRQOL in this population [23,24]. However, the primary objective of the present study was not to measure global HRQOL but to evaluate depressive symptoms using a validated, depression-focused instrument.

An important observation in our study was that no statistically significant associations were detected between depressive symptoms and LSM, UDCA treatment response, prognostic scores, and disease duration in patients with PBC. The relationship between depressive symptoms and liver disease severity in patients with PBC remains unclear. These findings cannot establish the independence of these PROs from liver disease severity, given the predominantly compensated cohort and only 12 patients with compensated cirrhosis. The limited liver disease severity range and sample size must be considered when comparing them with previous studies. Previous studies have shown that patients with PBC may experience substantial symptom burden, including depressive symptoms, fatigue, and impaired QoL, even in the earlier stages of the disease. In this context, fatigue and pruritus have been reported to exert major negative effects on emotional well-being, daily functioning, and overall HRQOL and may contribute to increased depression symptoms, independent of liver disease severity [20,21]. However, other studies have found an association between depressive symptoms and liver disease severity [11,18,23]. Several methodological issues may account for these inconsistencies. First, the definition of depression differs substantially among studies. Self-reported symptom scales may capture somatic symptoms, fatigue, sleep disturbance, and reduced activity, whereas structured psychiatric interviews more specifically identify major depressive disorder. Second, cohorts differ in disease stage: studies restricted to well-compensated or predominantly early-stage PBC may be less likely to detect an association with bilirubin, albumin, fibrosis stage, or prognostic scores, whereas cohorts including patients with Child–Pugh B/C disease may show stronger links between depressive symptoms and liver dysfunction. Third, ethnicity and cultural differences may influence symptom reporting, coping strategies, social support, and thresholds for reporting depressive symptoms. Finally, sample size and the inclusion or exclusion of patients with comorbidities are important because fatigue, sleep disturbance, autoimmune comorbidities, and pruritus may mediate depressive symptoms independently of liver disease severity and may obscure direct associations with biochemical and prognostic markers.

Sleep disturbance was another key finding of our study, as 62% of the patients had poor sleep quality, and there was a significant positive correlation between the BDI and PSQI scores. Sleep disturbance is increasingly recognized as an important contributor to the symptom burden of PBC. Compared with HCs, patients with PBC experience higher rates of fatigue, excessive daytime sleepiness, and poor sleep quality. Fatigue is highly prevalent, reported in the majority of patients, and is particularly pronounced in patients with advanced liver disease [20,25,26]. Notably, sleep impairment is closely intertwined with depressive and anxiety symptoms, suggesting a shared neurobiological and psychosocial pathway that contributes to the overall symptom burden in patients with PBC [27]. Pruritus may further contribute to nocturnal symptoms, as greater itch severity is associated with poor sleep quality and prolonged sleep latency [28]. Proposed mechanisms are multifactorial and include autonomic dysfunction, neurotransmitter alterations, neuromuscular abnormalities, and disruption of normal sleep–wake cycle regulation [29]. Our findings are consistent with previous studies that have demonstrated impaired sleep quality in patients with PBC [27,30]. Although excessive daytime somnolence has been reported in prior studies, our study did not evaluate PSQI subcomponents [26]. In a recent study, sleep disorders and impaired sleep patterns were associated with higher disease severity and worse clinical outcomes in patients with PBC [30]. Another study reported that patients with PBC with poor sleep quality had lower albumin levels [27]. Nonetheless, it remains uncertain whether sleep disorders are the cause or the result of this association. In our study, younger age was associated with poor sleep quality after adjustment for the GLOBE score and disease duration, suggesting that factors other than disease severity may influence sleep disturbance.

Anhedonia was another domain evaluated in our study, and to our knowledge, this is the first study to specifically assess anhedonia in patients with PBC. Using a SHAPS cutoff value of ≥3, clinically relevant anhedonia was identified in 36% of patients with PBC and 18% of HCs. In addition, SHAPS scores were significantly correlated with both BDI and PSQI scores. Anhedonia is increasingly recognized as a clinically relevant symptom of a wide range of chronic medical and psychiatric disorders. Although classically regarded as a core feature of depression, anhedonia is now understood as a broader disturbance in reward processing that may occur independently of depressive syndromes. It is also closely linked to fatigue, with emerging evidence suggesting overlapping biological pathways and frequent co-occurrence, particularly in chronic diseases [31,32]. The observed correlations between depressive symptoms, sleep disturbances, and anhedonia demonstrate co-occurrence but cannot establish the direction of relationships between these factors. Sleep disturbances may exacerbate depressive symptoms, and depression can impair sleep regulation. In addition, anhedonia may reflect depressive symptoms; however, the underlying mechanisms of these symptoms in patients with PBC remain unknown. Current hypotheses regarding depression in patients with PBC include disrupted neurotransmitter synthesis, alterations in brain structure observed through imaging, or a direct result of the symptom burden of fatigue and pruritus. Because fatigue was not measured, the study could not distinguish anhedonia from fatigue or quantify their overlap; the SHAPS findings should therefore not be interpreted as demonstrating a distinct PBC-specific symptom mechanism.

This study has several strengths, including the simultaneous assessment of depressive symptoms, sleep quality, and anhedonia using validated self-reported instruments; the inclusion of an HC group; and the concurrent evaluation of liver fibrosis and steatosis, UDCA treatment response, and established prognostic scores. Nevertheless, several limitations of this study should be acknowledged.

This single-center study had a relatively small sample size and limited representation of advanced liver disease: only 12 patients had clinical cirrhosis and 17 were classified as high risk for poor prognosis. Restricted liver disease severity range and wide confidence intervals may have obscured associations; non-significant findings are not evidence of equivalence or independence from liver disease severity. The cross-sectional design precluded causal or temporal conclusions. Fatigue and pruritus were not measured, preventing quantification of their contributions to mood, sleep, and anhedonia; nocturnal itch and fatigue-related daytime dysfunction may partly account for questionnaire scores. Anxiety was not evaluated with a dedicated instrument, and thyroid disease, Sjögren’s syndrome, menopausal and educational status were unavailable. HCs were selected to have no known chronic disease, whereas patients had diabetes, hypertension, MASLD, and other comorbidities. Despite age and sex adjustment, differences may reflect chronic illness more generally rather than PBC specifically. Exclusion of previously diagnosed major depressive disorder may underestimate symptom burden in the broader PBC population. Depression was not evaluated using a structured or semi-structured psychiatric interview; therefore, the findings reflect the depressive symptom burden rather than a formally diagnosed depressive disorder. Without structured psychiatric interviews, the clinical significance of the higher prevalence of depressive symptoms in patients with PBC cannot be established. We did not use a PBC-specific or dedicated health-related quality of life instrument, such as the PBC-40 or CLDQ-PBC; therefore, overall HRQOL could not be directly quantified.

## 5. Conclusions

Depressive symptoms, poor sleep quality, and anhedonia were common and interrelated in this PBC cohort and were more frequent than in selected HCs, including after adjustment for age and sex. No statistically significant associations were detected with the assessed liver disease severity and prognostic markers in this predominantly compensated cohort. Future studies should assess impaired HRQOL in patients with PBC with a broader range of disease severity and also include the assessment of fatigue, pruritus, anxiety, and detailed comorbidities. Clinical evaluation of these symptom domains, with psychiatric assessment when indicated, may help identify patients requiring further support.

## Figures and Tables

**Table 1:** Clinical characteristics of patients with primary biliary cholangitis.

Variables	*n* = 103
Age, years	57.2 ± 12.3
Sex, male	11 (10.7)
BMI, kg/m^2^	28.5 ± 4.8
Cirrhosis	12 (11.7)
CAP, dB/m	224 ± 45
LSM, kPa	7.6 (2.8–73.9)
ALT, U/L	21 (5–137)
AST, U/L	22 (11–120)
Alkaline phosphatase, U/L	134 (27–542)
Total bilirubin, mg/dL	0.49 (0.18–4.98)
Platelet count, ×10^3^/mm^3^	250 ± 84
MASLD	26 (25.2)
Diabetes	23 (22.3)
Hypertension	41 (39.8)
CMR factors ≥ 3	12 (11.7)
GLOBE score	−0.43 ± 0.84
GLOBE high-risk category	17 (17.2)
Paris II response (no)	33 (32)

Data are presented as *n* (%), mean ± SD, or median (minimum-maximum). BMI, body mass index; CAP, controlled attenuation parameter; LSM, liver stiffness measurement; MASLD, metabolic dysfunction-associated steatotic liver disease; CMR, cardiometabolic risk.

**Table 2:** Univariate and multivariate logistic regression analyses of moderate-to-severe depressive symptoms in patients with PBC.

	Univariate		Multivariate	
Variable	OR (95% CI)	*p*	OR (95% CI)	*p*
Age, per year	0.988 (0.957–1.019)	0.440	—	—
Male vs. female	0.427 (0.107–1.713)	0.230	0.343 (0.079–1.490)	0.153
BMI, per kg/m^2^	0.980 (0.904–1.063)	0.633	—	—
Clinical cirrhosis, yes vs. no	0.871 (0.257–2.950)	0.824	—	—
GLOBE score, per point	0.783 (0.486–1.264)	0.317	—	—
Paris II non-response vs. response	1.048 (0.456–2.409)	0.911	—	—
CAP ≥ 248 vs. <248 dB/m	1.103 (0.461–2.637)	0.825	—	—
LSM ≥ 8 vs. <8 kPa	1.736 (0.790–3.812)	0.170	2.040 (0.889–4.683)	0.093
Diabetes, yes vs. no	0.747 (0.290–1.924)	0.546	—	—
Hypertension, yes vs. no	1.554 (0.702–3.442)	0.277	—	—
ALP, per U/L	0.999 (0.995–1.003)	0.690	—	—
Total bilirubin, per mg/dL	0.869 (0.480–1.573)	0.643	—	—
MASLD, yes vs. no	1.084 (0.444–2.647)	0.859	—	—
Disease duration, per month	1.008 (0.998–1.018)	0.126	1.008 (0.998–1.018)	0.136

BMI, body mass index; CAP, controlled attenuation parameter; LSM, liver stiffness measurement; MASLD, metabolic dysfunction-associated steatotic liver disease. OR, odds ratio; CI, confidence interval.

**Table 3:** Univariable and multivariable logistic regression analyses of poor sleep quality in patients with PBC.

	Univariate		Multivariate	
Variable	OR (95% CI)	*p*	(95% CI)	*p*
Age, per year	0.958 (0.925–0.993)	0.020	0.946 (0.898–0.997)	0.039
Male vs. female	0.703 (0.200–2.480)	0.584	—	—
BMI, per kg/m^2^	1.046 (0.961–1.139)	0.299	—	—
Clinical cirrhosis, yes vs. no	0.835 (0.246–2.839)	0.773	—	—
GLOBE score, per point	0.734 (0.443–1.216)	0.230	1.221 (0.601–2.480)	0.581
Paris II non-response vs. response	0.909 (0.389–2.127)	0.826	—	—
CAP ≥ 248 vs. <248 dB/m	0.921 (0.378–2.245)	0.856	—	—
LSM ≥ 8 vs. <8 kPa	1.072 (0.481–2.391)	0.865	—	—
Diabetes, yes vs. no	1.524 (0.564–4.119)	0.406	—	—
Hypertension, yes vs. no	0.921 (0.409–2.075)	0.844	—	—
ALP, per U/L	1.000 (0.996–1.004)	0.854	—	—
Total bilirubin, per mg/dL	0.907 (0.513–1.602)	0.736	—	—
MASLD, yes vs. no	0.967 (0.387–2.413)	0.942	—	—
Disease duration, per month	1.006 (0.996–1.017)	0.226	1.006 (0.995–1.017)	0.305

BMI, body mass index; CAP, controlled attenuation parameter; LSM, liver stiffness measurement; MASLD, metabolic dysfunction-associated steatotic liver disease. OR, odds ratio; CI, confidence interval.

**Table 4:** Univariable logistic regression analyses of anhedonia in patients with PBC.

Variable	OR (95% CI)	*p*
Age, per year	0.986 (0.954–1.019)	0.412
Male vs. female	0.362 (0.074–1.773)	0.210
BMI, per kg/m^2^	1.024 (0.942–1.113)	0.579
Clinical cirrhosis, yes vs. no	1.317 (0.387–4.486)	0.660
GLOBE score, per point	0.878 (0.539–1.433)	0.603
Paris II non-response vs. response	1.506 (0.642–3.529)	0.346
CAP ≥ 248 vs. <248 dB/m	1.219 (0.498–2.985)	0.664
LSM ≥ 8 vs. <8 kPa	1.084 (0.483–2.435)	0.844
Diabetes, yes vs. no	0.729 (0.269–1.976)	0.535
Hypertension, yes vs. no	1.049 (0.461–2.385)	0.909
ALP, per U/L	1.002 (0.997–1.006)	0.464
Total bilirubin, per mg/dL	1.164 (0.658–2.059)	0.602
MASLD, yes vs. no	0.926 (0.365–2.353)	0.872
Disease duration, per month	1.006 (0.996–1.016)	0.260

BMI, body mass index; CAP, controlled attenuation parameter; LSM, liver stiffness measurement; MASLD, metabolic dysfunction-associated steatotic liver disease. OR, odds ratio; CI, confidence interval.

## Data Availability

The original contributions presented in this study are included in the article/Supplementary Material. Further inquiries can be directed to the corresponding author.

## Supplementary Materials

The following supporting information can be downloaded at https://www.mdpi.com/article/10.3390/jcm15207967/s1: STROBE Checklist. Table S1: Comparison of clinical parameters according to depressive symptom severity in patients with PBC; Table S2: Comparison of clinical parameters according to sleep quality in patients with PBC; Table S3: Comparison of clinical parameters according to anhedonia in patients with PBC; Table S4: Age- and sex-adjusted PBC vs healthy control comparisons of binary outcomes; Table S5: Age- and sex-adjusted PBC vs. healthy control continuous-score differences; Table S6: Associations of BDI score with liver disease severity markers in patients with PBC; Table S7: Associations of PSQI score with liver disease severity markers in patients with PBC; Table S8: Associations of SHAPS score with liver disease severity markers in patients with PBC.
