# Supplementary material for: Depressive Symptoms, Sleep Quality, and Anhedonia in Patients with Primary Biliary Cholangitis: A Cross-Sectional Study
Source: J Clin Med. 2026 Oct 15;15(20):7967. doi: 10.3390/jcm15207967 (PMC13706577; doi:10.3390/jcm15207967)
Supplement: Supplementary file 1 [file jcm-15-07967-s001.zip › STROBE_Checklist.pdf]

STROBE Checklist

Item	Reporting topic	Location / evidence	Status / qualification
1a	Design in title	Title; Abstract	Reported
1b	Balanced abstract	Abstract	Reported
2	Scientific rationale	Introduction, paragraphs 1–4	Reported
3	Objectives	Introduction, final paragraph	Reported
4	Design	Methods: Study participants, first paragraph	Reported
5	Setting and dates	Methods: Study participants; February–June 2025	Reported
6a	Eligibility and recruitment	Methods: Study participants; patient and control selection	Reported
7	Variables and definitions	Methods: Study participants; Questionnaires; Statistical analysis	Reported
8	Measurements	Methods: laboratory timing, VCTE protocol, questionnaires and clinical definitions	Reported
9	Bias	Methods: consecutive screening and age/sex adjustment; Discussion: selection and residual confounding	Reported
10	Study size	Results: 140 patients screened, 103 included, 50 controls	all eligible consecutive patients with PBC evaluated during the predefined study period were screened; no formal a priori sample-size calculation was performed.
11	Quantitative variables	Methods: Statistical analysis and Questionnaires	Reported; BDI ≥19, PSQI >5, SHAPS ≥3; predictor units in Tables 2–4
12a	Analysis and confounding	Methods: Statistical analysis; Tables 2–4 and S4–S8	Reported
12b	Subgroups and interactions	PBC-only models, Tables 2–4 and S6–S8	PBC subgroup reported
12c	Missing data handling	Methods: complete cases without imputation; Results; Table S10	Reported
12d	Sampling analysis	Methods: consecutive patients and	Reported

		institutional volunteers	
12e	Sensitivity analyses	Methods; Tables S5–S8	Exploratory continuous-score analyses reported
13a	Participant numbers	Results: Characteristics of participants	Reported
13b	Nonparticipation	Results: 37 exclusions with reasons	Reported
13c	Flow diagram	Results: textual screening flow	Counts described in text
14a	Participant characteristics	Results: Characteristics of participants; Table 1; Tables S1–S3	Reported
14b	Missingness per variable	Results; Table S10; Table 1 cirrhotic subgroup	Reported
15	Outcome frequencies	Abstract; Results: questionnaire comparison and category analyses	Reported
16a	Estimates and precision	Tables 2–4 and S4–S8; Results	Unadjusted and adjusted effects, 95% CIs, and adjustment variables reported
16b	Category boundaries	Methods: Questionnaires and Statistical analysis	Reported
16c	Absolute risk translation	Not applicable	Cross-sectional prevalence study; no time-based risk estimate
17	Additional analyses	Correlation Results; Tables S5–S9	Reported
18	Key findings	Discussion, opening paragraph; Conclusion	Reported
19	Limitations	Discussion: limitations paragraph	Reported, including restricted severity, symptom omissions, healthy-control selection and missing scores
20	Cautious interpretation	Abstract conclusion; Discussion; Conclusion	Reported; no causal or independence claim; exploratory multiplicity acknowledged
21	Generalizability	Discussion: limitations paragraph	Reported; single center, selected controls and predominantly compensated patients
22	Funding and funder role	Funding statement	No specific funding was received for this study.
