# Supplementary material for: Depressive Symptoms, Sleep Quality, and Anhedonia in Patients with Primary Biliary Cholangitis: A Cross-Sectional Study
Source: J Clin Med. 2026 Oct 15;15(20):7967. doi: 10.3390/jcm15207967 (PMC13706577; doi:10.3390/jcm15207967)
Supplement: Supplementary file 1 [file jcm-15-07967-s001.zip › Supplementary_Revised.pdf]

Supplementary Material

Depressive symptoms, sleep quality, and anhedonia in patients with primary biliary cholangitis: a cross-sectional study

Table S1. Comparison of clinical parameters according to depressive symptom severity in patients with PBC

Variable	Moderate-to-severe depressive symptoms (n=46)	Minimal or mild depressive symptoms (n=57)	p
**Age, years**	56 ± 14	58 ± 11	0.444
**Sex, male**	3 (6.5)	8 (14)	0.338
**BMI, kg/m²**	28.3 ± 4.7	28.7 ± 4.9	0.636
**CAP score ≥ 248 dB/m**	13 (28.3)	15 (26.3)	0.825
**LSM ≥ 8, kPa**	24 (52.2)	22 (38.6)	0.168
**Cirrhosis**	5 (10.9)	7 (12.3)	0.824
**MASLD**	12 (26.1)	14 (24.6)	0.859
**Diabetes**	9 (19.6)	14 (24.6)	0.545
**Hypertension**	21 (45.7)	20 (35.1)	0.276
**GLOBE score**	−0.53 ± 0.95	−0.35 ± 0.74	0.320
**Paris II response (no)**	15 (32.6)	18 (31.6)	0.911
**Disease duration, months**	80.4 ± 41.2	68.1 ± 39.1	0.125

Data are presented as n (%) or mean ± SD. BMI, body mass index; CAP, controlled attenuation parameter; LSM, liver stiffness measurement; MASLD, metabolic dysfunction-associated steatotic liver disease.

Table S2. Comparison of clinical parameters according to sleep quality in patients with PBC

Variable	Poor sleep quality (n=64)	Good sleep quality (n=39)	p
**Age, years**	54.9 ± 12.9	60.9 ± 10.3	0.017
**Sex, male**	6 (9.4)	5 (12.8)	0.744
**BMI, kg/m²**	28.9 ± 4.9	27.9 ± 4.6	0.301
**CAP score ≥ 248 dB/m**	17 (26.6)	11 (28.2)	0.856
**LSM ≥ 8, kPa**	29 (45.3)	17 (43.6)	0.865
**Cirrhosis**	7 (10.9)	5 (12.8)	0.762
**MASLD**	16 (25)	10 (25.6)	0.942
**Diabetes**	16 (25.0)	7 (17.9)	0.405
**Hypertension**	25 (39.1)	16 (41)	0.843
**GLOBE score**	−0.51 ± 0.89	−0.30 ± 0.75	0.232
**Paris II response (no)**	20 (31.3)	13 (33.3)	0.826
**Disease duration, months**	77.4 ± 43.3	67.5 ± 34.6	0.228

Data are presented as n (%) or mean ± SD. BMI, body mass index; CAP, controlled attenuation parameter; LSM, liver stiffness measurement; MASLD, metabolic dysfunction-associated steatotic liver disease.

Table S3. Comparison of clinical parameters according to anhedonia in patients with PBC

Variable	Anhedonia (n=37)	No anhedonia (n=66)	p
**Age, years**	55.9 ± 14.7	57.9 ± 10.8	0.415
**Sex, male**	2 (5.4)	9 (13.6)	0.320
**BMI, kg/m²**	28.8 ± 4.7	28.3 ± 4.9	0.582
**CAP score ≥ 248 dB/m**	11 (29.7)	17 (25.8)	0.664
**LSM ≥ 8, kPa**	17 (45.9)	29 (43.9)	0.844
**Cirrhosis**	5 (13.5)	7 (10.6)	0.752
**MASLD**	9 (24.3)	17 (25.9)	0.872
**Diabetes**	7 (18.9)	16 (24.2)	0.534
**Hypertension**	15 (40.5)	26 (39.4)	0.909
**GLOBE score**	−0.49 ± 1.0	−0.39 ± 0.73	0.607
**Paris II response (no)**	14 (37.8)	19 (28.8)	0.345
**Disease duration, months**	79.5 ± 42.8	70.2 ± 38.7	0.262

Data are presented as n (%) or mean ± SD. BMI, body mass index; CAP, controlled attenuation parameter; LSM, liver stiffness measurement; MASLD, metabolic dysfunction-associated steatotic liver disease.

Table S4. Age- and sex-adjusted PBC vs healthy control comparisons of binary outcomes

Outcome	PBC vs healthy control OR (95% CI)	p
**Moderate-to-severe depressive symptoms**	3.889 (1.695–8.926)	0.001
**Poor sleep quality**	3.442 (1.636–7.238)	0.001
**Anhedonia**	2.678 (1.158–6.193)	0.021

Logistic regression adjusted for age and sex. OR, odds ratio; CI, confidence interval.

Table S5. Age- and sex-adjusted PBC vs. healthy control continuous-score differences

Questionaries	Adjusted score difference (95% CI)	p
**BDI**	6.370 (3.174–9.565)	<0.001
**PSQI**	1.823 (0.778–2.869)	<0.001
**SHAPS**	1.205 (0.253–2.157)	0.013

Positive coefficients indicate higher scores in patients with PBC. Linear models adjusted for age and sex used HC3 robust standard errors.

Table S6. Associations of BDI score with liver disease severity markers in patients with PBC

Variable	Age-/sex-adjusted coefficient (95% CI)	p
**Continuous LSM, per kPa**	-0.025 (-0.406–0.355)	0.895
**Clinical cirrhosis, yes vs no**	-2.154 (-12.656–8.349)	0.685
**GLOBE score, per point**	-0.162 (-3.392–3.069)	0.921
**Paris II non-response vs response**	1.811 (-4.726–8.347)	0.583
**ALP, per U/L**	0.0066 (-0.0353–0.0485)	0.755
**Total bilirubin, per mg/dL**	1.038 (-8.654–10.730)	0.832

HC3 robust standard errors were used. CI, confidence interval.

Table S7. Associations of PSQI score with liver disease severity markers in patients with PBC

Variable	Age-/sex-adjusted coefficient (95% CI)	p
**Continuous LSM, per kPa**	-0.016 (-0.102–0.069)	0.706
**Clinical cirrhosis, yes vs no**	-0.046 (-3.687–3.596)	0.980
**GLOBE score, per point**	0.160 (-1.042–1.361)	0.792
**Paris II non-response vs response**	-0.403 (-2.191–1.386)	0.656
**ALP, per U/L**	-0.0040 (-0.0149–0.0068)	0.462
**Total bilirubin, per mg/dL**	0.190 (-1.147–1.528)	0.778

HC3 robust standard errors were used. CI, confidence interval.

Table S8. Associations of SHAPS score with liver disease severity markers in patients with PBC

Variable	Age-/sex-adjusted coefficient (95% CI)	p
**Continuous LSM, per kPa**	0.027 (-0.095–0.149)	0.661
**Clinical cirrhosis, yes vs no**	0.050 (-3.308–3.409)	0.976
**GLOBE score, per point**	0.778 (-0.693–2.250)	0.296
**Paris II non-response vs response**	1.730 (-0.407–3.866)	0.111
**ALP, per U/L**	0.0054 (-0.0031–0.0140)	0.211
**Total bilirubin, per mg/dL**	0.695 (-0.563–1.953)	0.275

HC3 robust standard errors were used. CI, confidence interval.
